# Influence of nicotine concentration and flavours on mouth level exposure and puffing topography among regular e-cigarette consumers in New Zealand

**DOI:** 10.1038/s41598-026-53889-3

**Published:** 2026-05-30

**Authors:** Lauren Edward, Krishna Prasad, Linsey E. Haswell, Adam Gray, Tahseen Jilani, Stacy Fiebelkorn

**Affiliations:** 1 BAT (Investments) Limited, Regents Park Road, Millbrook, Southampton, SO15 8TL UK; 2 Lilac Grove Consultancy, Dubai, United Arab Emirates

**Keywords:** Average daily consumption (ADC), Bridging, Puffing topography, Electronic nicotine delivery system, ENDS, Electronic cigarette, Vuse, Mouth level exposure (MLE), Use behaviour, Biomarkers, Chemistry

## Abstract

E-cigarette aerosols have substantially reduced toxicant profiles relative to cigarette smoke. Consumers’ actual exposure is influenced by the way in which they use these products. We have assessed whether consumer puffing topography (e.g., puff duration, volume and frequency), mouth level exposure (MLE) to nicotine and aerosol, or average daily consumption (ADC) are affected by nicotine concentration or flavour. The study was conducted in New Zealand in 2023 among 90 adult e-cigarette users. Puffing topography and MLE were measured for eight e-liquid variants (five nicotine levels, four flavours) in (1) 15-minute vaping sessions during central location testing (CLT); and (2) 7-day home use testing (HUT). The CLT estimated MLE to nicotine increased with increasing concentration of nicotine, while MLE to aerosol decreased, puff duration decreased and interpuff interval increased, indicating that users alter puffing behaviour to achieve a preferred level of nicotine. Results from the HUT supported these findings. In CLT, less acceptable flavours (e.g., Golden Tobacco) led to significantly fewer puffs of shorter duration compared with more acceptable flavours (e.g., Crisp Mint and Tropical Mango). When the participants used their preferred flavour in HUT, there were no differences in MLE per day or ADC among flavours. E-cigarette users maintain a preferred level of nicotine exposure per day by altering the duration and number of puffs taken in response to nicotine concentration. Overall liking of a flavour is likely to be important in providing a satisfactory alternative to continued smoking for smokers who would not otherwise quit.

**Supplementary Information:**

The online version contains supplementary material available at 10.1038/s41598-026-53889-3.

##  Introduction

The health risks associated with smoking, primarily resulting from the long-term inhalation of tobacco smoke containing multiple toxicants^1^, are well established^2^. Electronic nicotine delivery systems (ENDS) also known as electronic cigarettes (e-cigarettes) provide a nicotine-containing aerosol with significantly fewer and substantially lower levels of toxicants than are found in cigarette smoke^3–8^, leading to reduced toxicological effects (e.g^8–11^). As a result, some health authorities support the use of ENDS as an alternative to smoking conventional cigarettes as part of a tobacco harm reduction (THR) strategy, which aims to help those who would not otherwise quit smoking to instead use a less harmful alternative product^12–16^. The validity of THR is supported by the findings of several cross-sectional and longitudinal studies^17–19^.

In addition to demonstrating reduced toxicant emissions relative to tobacco cigarettes, it is essential to determine how consumers are using ENDS, since the way in which they are used, such as use frequency and puff duration, will determine a consumer’s actual exposure to toxicants and other constituents of interest. Recently, multi-disciplinary assessment framework approaches, including a 9-step comprehensive scientific weight of evidence evaluation outlined by^20^; have been utilised to establish the reduced risk potential of ENDS and other alternative tobacco and nicotine products when compared to cigarettes^20–23^. These assessment frameworks comprise of measurements of aerosol emission constituents, consumer use behaviour and exposure, pre-clinical and clinical studies, post-market surveillance and epidemiological modelling^20^. Consumer use behaviour studies measure puffing topography and estimated mouth level exposure (MLE) to nicotine and other aerosol constituents of inhaled nicotine and tobacco products when used by consumers, playing a key role in verifying that consumers use the product in a manner that results in reduced individual exposure relative to combustible cigarettes^24,25^. Puffing topography is the pattern of behaviour observed when consumers draw on aerosol products and includes parameters such as puff duration, puff volume, interpuff interval (IPI; the time between puffs), flow rate and puff number, which are then used to estimate MLE to aerosol constituents including nicotine^24–26^.

Puffing topography data are also important for informing emissions analyses and hence for regulatory purposes^27^. Currently, standardised laboratory machine puffing regimes (ISO, CORESTA) are used to collect and analyse ENDS aerosol^28,29^ and their use allows comparisons among different studies and products; however, these regimes do not necessarily reflect real-world puffing behaviour. Puffing topography studies help to characterise consumer behaviour and exposure, informing the extent to which current machine puffing regimes are reflective of actual use and enabling better estimates of potential harm.

Different approaches have been taken to obtain puffing topography data for ENDS: for example, location-based studies require participants to visit a central location and use the study products either through a puffing analyser^24,25^ or while being video recorded^30^, while the recent development of connected devices has allowed the acquisition of data while participants use the products in their everyday environments^31–34^. These studies have identified several factors that affect consumer use behaviour of ENDS, including e-liquid flavour, nicotine concentration, coil resistance, power setting and pressure drop, with higher power settings leading to shorter puff durations and devices of lower open pressure drop leading to larger puff volumes^26,35–39^. Regarding nicotine in particular, consumers have been reported to take longer puff durations and consume more e-liquid when using products with lower nicotine concentrations^40–42^, showing compensatory puffing behaviour to achieve a preferred level of nicotine, as is well documented in cigarette smoking^43^. Notably, however, many puffing topography studies have been small-scale or pilot studies, with low numbers of participants and few conditions compared (e.g., high vs. low nicotine; high vs. low power).

In the present study, we measured puffing topography, MLE and average daily consumption (ADC) among 90 regular e-cigarette (ENDS) consumers using a specific ENDS device with e-liquids of five nicotine concentrations (6–47 mg/mL) and four flavours, in order to create a foundational dataset for consumer use behaviour of ENDS. For comparison, puffing topography and MLE were also measured among 35 regular smokers using a 15 mg tar combustible cigarette.

## Materials and methods

### Study design

This cross-sectional controlled-use study of puffing topography was conducted in New Zealand between January and March in 2023, among current users of ENDS and combustible cigarettes, in two locations, a standardised test centre (in Christchurch, New Zealand) and participants’ home environments. The study was run onsite in Christchurch by BAT staff and local agency staff.

All participants provided written informed consent prior to undergoing any study procedures. The study protocol and informed consent form were approved by the independent ethics committee (IEC) of Cerner Enviza in accordance with the ethical principles outlined in the Declaration of Helsinki.

### Study products

Eight Vuse vPro e-liquids (BAT, London, UK) across a range of nicotine concentrations (6–47 mg/mL) and flavour categories (mint, tobacco and fruit) were used with the same Vuse ePod 2 devices (BAT) (Table 1, products 1–8). Vuse ePod 2 is a puff-activated closed-system ENDS consisting of a rechargeable battery section and a non-refillable e-liquid cartridge. The pre-filled pod (1.9 mL) is magnetically attached to the battery section and heats the nicotine-containing e-liquid using a ceramic wick and flat metal heating element, producing an aerosol which is inhaled into the user’s mouth during puffing. A 15-mg ISO tar cigarette was also included (Rothmans Royal, BAT) (Table 1, product 9) and used by the regular smokers only. All study products were commercially available in New Zealand during the time of the study.

**Table 1:** Study products.

Product	Product name/type	Product description	Nicotine strength
1	Vuse ePod 2	vPro Crisp Mint	6 mg/mL (0.5%)
2	Vuse ePod 2	vPro Crisp Mint	12 mg/mL (1%)
3	Vuse ePod 2	vPro Crisp Mint	18 mg/mL (1.6%)
4	Vuse ePod 2	vPro Crisp Mint	34 mg/mL (3%)
5	Vuse ePod 2	vPro Crisp Mint	47 mg/mL (4%)
6	Vuse ePod 2	vPro Golden Tobacco	18 mg/mL (1.6%)
7	Vuse ePod 2	vPro Tropical Mango	18 mg/mL (1.6%)
8	Vuse ePod 2	vPro Peppermint Tobacco	18 mg/mL (1.6%)
9	Combustible cigarette	Rothmans Royal	15 mg

### Study population

The study population was recruited from two sources, a New Zealand Vuse consumer database (managed by BAT) and a consumer panel run by an independent market research agency (Resona Research, New Zealand) in accordance with the International Code on Market Opinion and Social Research and Data Analytics^44^. A screening questionnaire was used to identify target respondents, who were regular healthy adult (21–65 years) e-cigarette (ENDS) users of 18, 34 or 47 mg/mL nicotine e-liquids (Study Arm 1), equally split between gender and nicotine use levels, and 35 regular cigarette smokers (Study Arm 2). For study arm 1, the inclusion criteria were (1) currently a regular vaper of a pod-based e-cigarette system in one of the above-mentioned nicotine strength e-liquids, and (2) using at least one cartridge per week for at least the past 6 months; and (3) willing to try tobacco, mint, peppermint tobacco and mango flavoured e-liquids. The inclusion criteria for Study Arm 2 additional inclusion criteria were (1) currently a regular smoker, smoking an average of 10 cigarettes per day for at least the past year, and (2) currently smoking Rothmans Royals. The exclusion criteria for both study arms were (1) pregnant or breastfeeding, or planning to start breastfeeding in the next three months, (2) having a pacemaker or other embedded electronic medical devices fitted; (3) employment (either themselves or close family/friends) in the fields of market research, marketing, advertising, public relations, journalism, media, insurance, manufacturer or retailer of tobacco or e-cigarettes, retailer of alcohol; (4) having taken part in any market research survey within 3 months prior to screening; and (5) planning to quit vaping or smoking in the next 6 months.

After providing consent, participants were allocated a unique volunteer ID code used to anonymously identify them in the study. They received remuneration for taking part in the study and were free to withdraw at any time.

### Study protocol

#### Central location testing

**Fig. 1:**
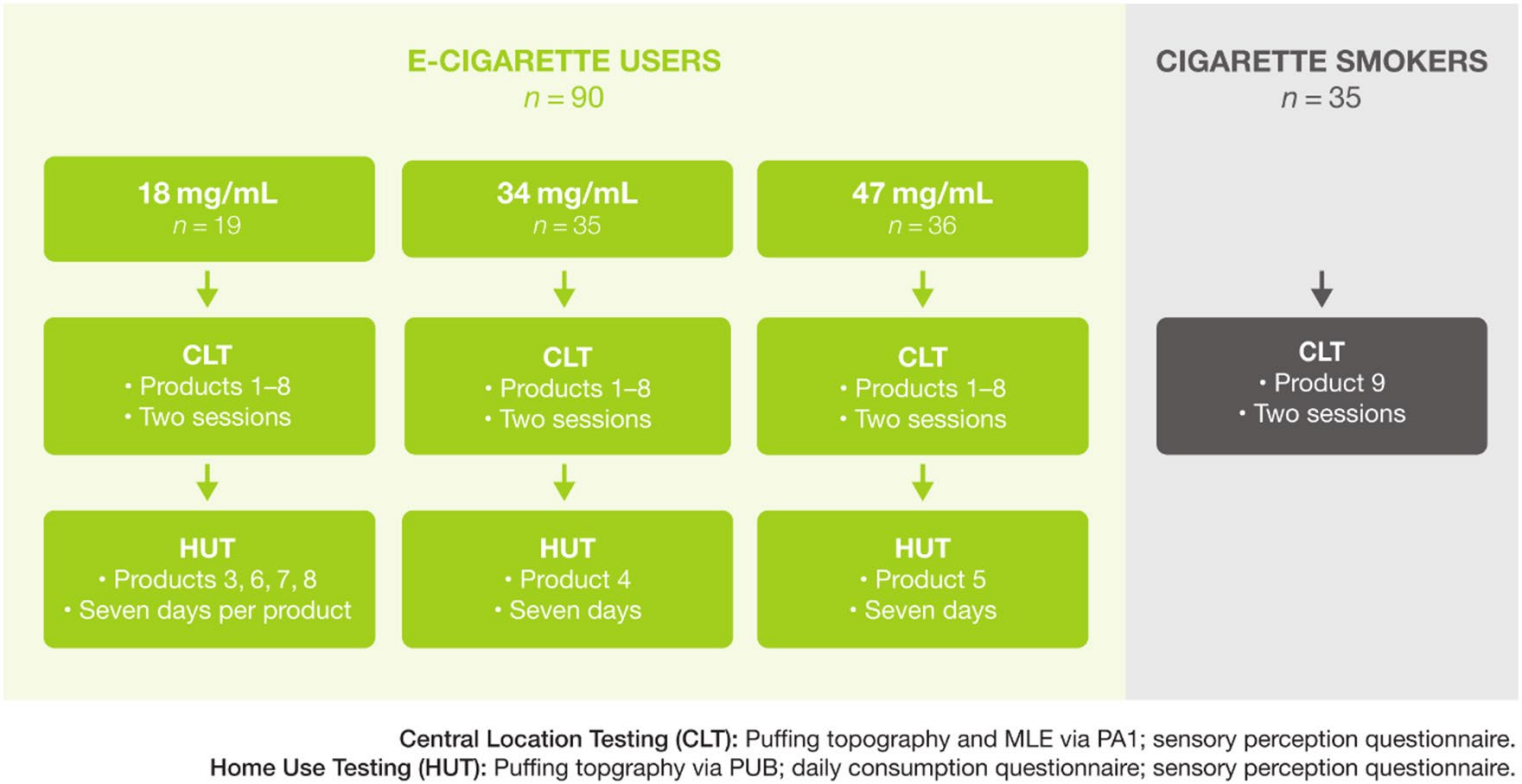
Summary of the study protocol.

Eligible study participants attended a central location testing (CLT) facility. Sociodemographic data, including ethnicity and age, were collected via a short online survey before puffing topography was measured using a desktop-based puffing analyser device (PA1, developed at BAT and manufactured by Turquoise Engineering)^24,25,45^. ENDS users attended the CLT facility for three separate visits to use the ENDS products (Fig. 1). They used up to three products per visit, with a minimum of 2 h between products and 24 h between visits. Products were pseudo-randomised such that the e-liquids with the highest nicotine concentrations (34 mg/mL and 47 mg/mL) were not used on the same visit (to manage nicotine exposure and ensure that regular 18 mg/mL nicotine users were not exposed to unusually high nicotine concentrations). During each product use session, participants used the allocated product for up to 15-minutes through the PA1 product holder, puffing as they would normally. Smokers attended the CLT facility for a single visit using only the 15 mg cigarette through the PA1, again puffing as they would normally (Fig. 1).

Two product use sessions were carried out per product, with a minimum 20-minutes break between the two sessions. A disposable plastic mouthpiece was attached to the PA1 product holder at the beginning of each session (to avoid cross contamination between participants). During the 20-minutes interval between product use repeats, participants completed an online sensory questionnaire, administered by Resona Research, which included questions on draw effort, intensity, aerosol delivery, amount of aerosol filling the mouth, irritation and taste on a magnitude scale ranging from 1 (low) to 5 (high).

####  Home use testing

After the CLT, participants in Study Arm 1 completed a home use testing (HUT) period. During the HUT period, the Vuse ePod 2 device was attached to a Product Use Behaviour (PUB) connected device (manufactured by Carolina Medical Electronics, North Carolina, USA)^33^, manufactured specifically for the Vuse ePod 2 in conjunction with a daily consumption survey, administered by Resona Research, to determine their puffing behaviour and ADC over 7 days. Participants who were regular users of 18 mg/mL nicotine products used each of the four flavours (Crisp Mint, Golden Tobacco, Peppermint Tobacco, Tropical Mango) at 18 mg/mL nicotine for a total of four consecutive 7-day HUT periods. Participants who were regular users of 34 or 47 mg/mL nicotine products used the Crisp Mint flavour at their usual nicotine concentration for one 7-day HUT period. Participants were provided with a Vuse ePod 2 PUB connected device and a 1-week supply of the allocated e-liquid cartridge (equivalent to their self-declared weekly e-liquid consumption at screening plus ~ 20%). They were also given an Android mobile phone (Samsung A21), providing access to an online daily consumption survey and sensory questionnaire, and an App for connecting to the PUB device and transferring the puffing behaviour data. The mobile phones were locked so that participants had access only to Wi-Fi, Bluetooth, questionnaires and the PUB transfer application. Participants were asked to use the PUB device attached to the allocated study product in place of their normal product for 7 days, completing the sensory questionnaire and consumption diary once per day to record their perception and consumption of the study product, as well as their use of any non-study products used during the HUT period.

### Measurements of puffing topography

At the CLT, topography measurements, including number of puffs taken, puff volume, puff duration, IPI and session duration, were recorded using a desktop-based puffing analyser device (PA1, formerly known as SA7^45^. The puffing analyser was originally developed to measure cigarette smoking topography, and later was modified for use with ENDS and heated tobacco products^24,25,45,46^. In brief, it comprises a product holder attached to a data acquisition transmission unit. Two tubes on either side of a 2-mm diameter orifice within the product holder detect the change in pressure during puffing, which is proportional to the flow rate squared^45^. Data are transferred to a laptop, which displays puff data in real time. During the CLT sessions, the puffing analyser device was operated by trained members of BAT staff.

For the HUT, puffing behaviour data were recorded by the PUB device, which measures the voltage, current, puff duration, number of puffs, IPI, angle held, and date and time of each puff taken by the consumer^33^. The device has a sleeve-like design that connects the battery section and e-liquid cartridge of the ENDS, enabling collection of data in consumers’ everyday environments. A session was defined for a given participant when the IPI for a given puff was greater than or equal to a threshold determined for that individual. The threshold value was determined as the 95th percentile of the participant’s overall IPI (excluding IPIs of 2 h or more). The IPI of the first and last puff in each session was not included in the calculation for mean IPI to avoid any effect of large time differences between sessions on the mean value. The puffing behaviour data gathered by the PUB device are stored in the instrument’s memory and then transferred by Bluetooth to a cloud database.

### Estimate of mouth level exposure

For the CLT, MLE to aerosol-collected mass (ACM), nicotine and menthol during the CLT was estimated from the device mass loss (DML) by using calibration graphs generated for each product under a range of machine puffing regimes, as described previously^25,26^. The resulting regression equations (R^2^ = 95.0%–99.0%) were used to estimate MLE to ACM, nicotine and menthol from the DML when the products were used by the study participants.

Mouth level exposure to nicotine and nicotine-free dry particulate matter (NFDPM) for the cigarette was estimated from real-time light obscuration in each puff measured by a light-emitting diode within the PA1 product holder, as previously described^24,25,45^.

For the HUT, estimates of MLE to ACM, nicotine and menthol were calculated from the puff duration measured by the PUB device, which was shown to be highly correlated with aerosol mass in laboratory testing (R^2^ = 97.3%; Supplementary Figure S1). Calibration graphs were obtained for a range of machine puffing regimes, and the resulting regression equations were used to estimate the MLE to ACM, nicotine and menthol per puff from the measured puff duration when the PUB device was used by the study participants.

###  Data analysis

This was a cross-sectional study, and no formal sample size calculation was conducted. Instead, the studied aimed to recruit 100 regular e-cigarette (ENDS) users. Data relating to participants who did not complete the study were excluded from the final analysis.

All analyses were performed in SAS v.9.4 statistical analysis software (SAS, Cary, NC, USA). Puffing topography, MLE, ADC and sensory perception responses are reported using mean ± standard deviation (SD) per product. Puffing topography and MLE data per product in CLT were first averaged for each participant across the two repeats. The product mean was then calculated as the mean of these participant-level averages. Puffing topography and MLE data per product in HUT were first averaged per participant for each day, then averaged across the 7-days. The mean per product was then calculated as the mean of these participant-level averages. Consumption data recorded in daily consumption diaries were entered by participants as free-text descriptions of pod proportions (e.g., “half a pod”, “3/4 of a pod”, “1 1/2 pods”). These entries were first converted to decimal values (e.g., 0.5, 0.75, 1.5). For each participant, daily values were averaged across the 7-day period. Final ADC per product was then calculated as the mean of these participant-level averages. A mixed-effects ANOVA with product as a fixed effect and subject as a random effect, followed by Tukey’s post-hoc test was used to analyse any differences in puffing topography, MLE and sensory perception among product data collected in the CLT, and among flavours used by 18 mg/mL nicotine users during the HUT. One-way ANOVA, followed by Tukey’s post hoc test was used to analyse any differences in puffing topography and MLE across user groups in the HUT. A two-sample t-test was used to analyse differences in puff number and MLE between the 47 mg/mL nicotine ENDS and the cigarette. A *P* value of less than 0.05 was taken to be statistically significant.

##  Results

###  Characteristics of the study population

The study recruited 97 regular ENDS users and 35 regular smokers. Seven ENDS users did not complete the study and were excluded from data analysis. The sociodemographic characteristics of the study population are summarized in Table 2. There were slightly more male ENDS users than female (54% vs. 46%), but a higher proportion of female smokers (60% vs. 40%). The ENDS users were predominantly in the age range 21–45 years with the highest proportion in the range 21–25 years (31%), whilst the highest proportion of smokers were in the age range 36–45 years (29%).

In both arms of the study, participants were predominantly New Zealand European (73% of ENDS users and 61% of smokers). Overall, 9% of ENDS users and 20% of smokers were Māori; these proportions increased to 16% and 26%, respectively, when participants with half Māori ethnicity were included. Of the ENDS users, 19 (21%) were regular users of 18 mg/mL nicotine products, 35 (39%) were regular users of 34 mg/mL, and 36 (40%) were regular users of 47 mg/mL products.

**Table 2:** Sociodemographic characteristics of the study participants.

Characteristic	ENDS users (*n* = 90)	Smokers (*n* = 35)
Gender		
Male	49 (54.4%)	14 (40.0%)
Female	41 (45.6%)	21 (60.0%)
Age group, years		
21–25	28 (31.1%)	4 (11.4%)
26–30	21 (23.3%)	7 (20.0%)
31–35	17 (19.0%)	5 (14.3%)
36–45	21 (23.3%)	10 (28.6%)
46–55	2 (2.2%)	7 (20.0%)
56–65	1 (1.1%)	2 (5.7%)
Ethnicity		
African English	1 (1.1%)	–
Asian	2 (2.2%)	–
Australian	1 (1.1%)	–
Caucasian	1 (1.1%)	–
European	2 (2.2%)	2 (5.7%)
Indian	–	1 (2.9%)
Māori	8 (9.0%)	7 (20.0%)
Māori/Asian	1 (1.1%)	–
Māori/New Zealand European	5 (5.6%)	2 (5.7%)
Middle Eastern	1 (1.1%)	–
New Zealand European	66 (73.3%)	21 (60.0%)
Pacific peoples ^a^	1 (1.1%)	2 (5.7%)
Pacific peoples/New Zealand European	1 (1.1%)	–

^a^ Includes Niuean and Samoan Pacific Peoples.

### Puffing topography and mouth level exposure

####  Effect of e-liquid nicotine concentration

To determine whether consumers’ puffing topography and MLE change in response to the nicotine concentration of the e-liquid, we conducted both CLT, where all 90 participants used Crisp Mint e-liquid at five nicotine levels in 15-minutes controlled vaping sessions, and HUT, where participants used the Crisp Mint e-liquid at their self-reported usual nicotine concentration (18, 34 or 47 mg/mL) in their normal environments for 7 days.

**Fig. 2:**
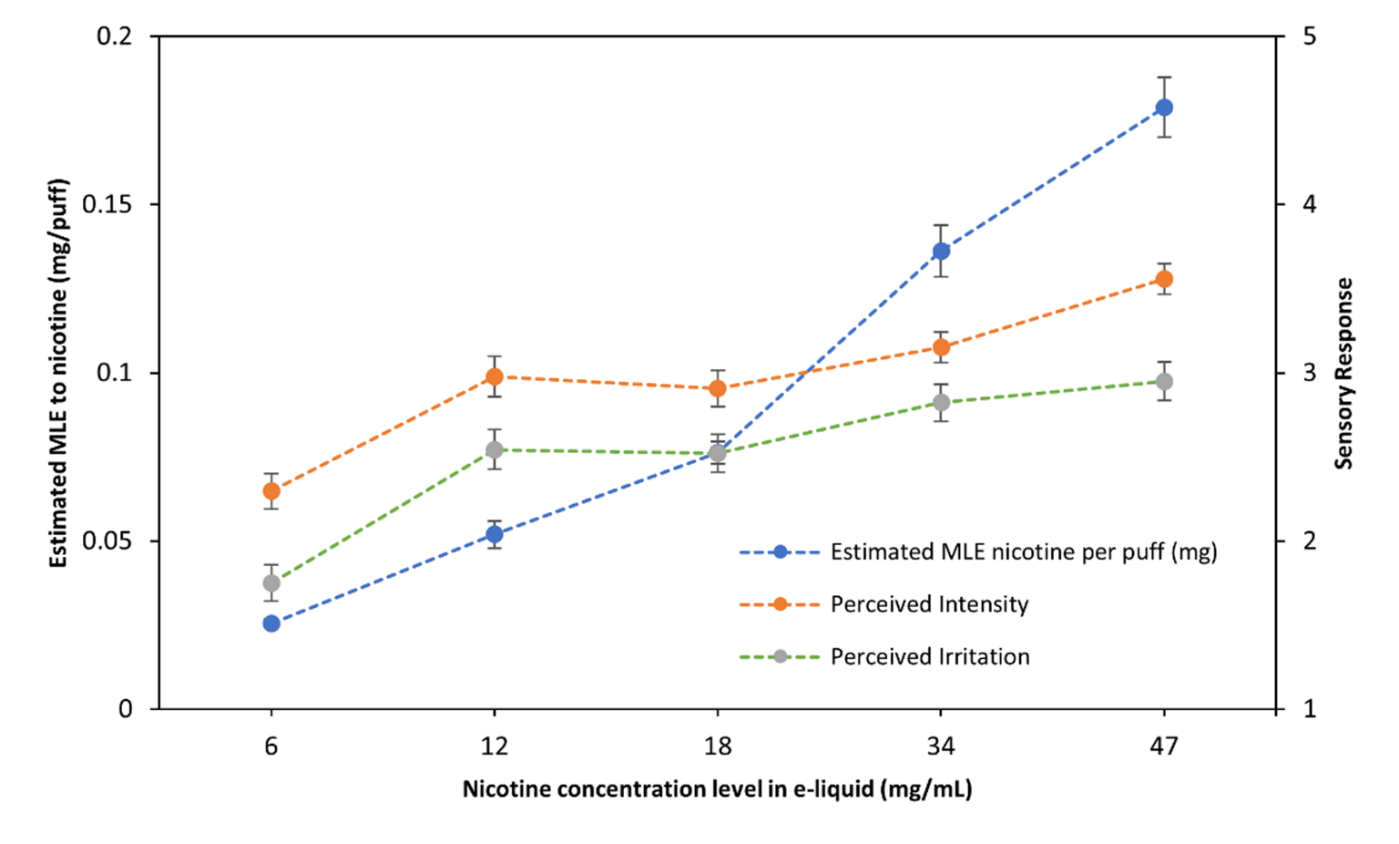
CLT: estimated MLE to nicotine per puff, and perceived intensity and irritation rating with increasing e-liquid nicotine concentration. Participants rated their perception of intensity and irritation on a scale of 1–5, where 1 was “low” and 5 was “high”. Error bars indicate standard error.

During CLT, MLE to nicotine (per puff and per session) increased with increasing concentration of nicotine in the e-liquid (0.74–4.07 mg nicotine per session) (Table 3) as expected. By contrast, MLE to total aerosol mass decreased with increasing concentration of nicotine (148.0–101.4 mg aerosol per session), consistent with participants generally taking slightly fewer puffs with shorter puff duration and reduced frequency as the nicotine concentration in the e-liquid increased. This was reflected in an increase in the perception of intensity and irritation by the study participants, although Overall liking was similar among the products (Fig. 2 and Supplementary Table S1). As a consequence of the MLE to aerosol mass decreasing with increasing nicotine concentration, the MLE to menthol also decreased (Table 3), and therefore it can be assumed that the MLE to other aerosol constituents (such as PG and VG) also decreased.

Whereas the differences in puffing parameters and MLE among the 6, 12 and 18 mg/mL e-liquids were small, those between the 34 and 47 mg/mL e-liquids were considerably more pronounced (118.3–101.4 mg aerosol per session for 34 and 47 mg/mL e-liquids vs. 148.0–139.8 mg aerosol per session for the 6, 12 and 18 mg/mL e-liquids).

To examine whether the effect of nicotine concentration differed according to participants’ usual nicotine concentration, the CLT puffing topography and MLE data were stratified by regular-use nicotine group (18, 34 and 47 mg/mL). These stratified data are presented as descriptive values in Supplementary Table S2. Overall, the same directional pattern of reduced puff duration and puff number, and increased IPI with increasing nicotine strength was observed across all user groups.

**Table 3:** CLT: comparison of puffing topography and MLE by nicotine concentration for Crisp Mint e-liquid^1^.

Parameter	6 mg/mL	12 mg/mL	18 mg/mL	34 mg/mL	47 mg/mL
Puff number^2^	30.3 ± 15.3^AB^	32.2 ± 15.3^A^	30.5 ± 18.0^AB^	28.6 ± 16.4^B^	24.9 ± 13.4^C^
Puff volume (mL)	52.3 ± 22.1^A^	49.8 ± 19.8^A^	49.5 ± 19.9^A^	50.1 ± 19.4^A^	45.5 ± 19.5^B^
Puff duration (s)	2.58 ± 0.93^A^	2.34 ± 0.87^B^	2.27 ± 0.89^BC^	2.18 ± 0.78^CD^	2.10 ± 0.78^D^
Interpuff interval (s)	35.1 ± 21.0^B^	36.1 ± 37.4^B^	35.3 ± 21.1^B^	38.6 ± 22.7^AB^	43.4 ± 26.3^A^
Effort (cmWGs)	953 ± 699^A^	869 ± 537^AB^	812 ± 602^BC^	737 ± 464^C^	614 ± 431^D^
MLE to aerosol per session (mg)	148.0 ± 101. 2^A^	147.2 ± 80.3^A^	139.8 ± 70.6^A^	118.3 ± 56.7^B^	101.4 ± 55.9^B^
MLE to nicotine per session (mg)	0.74 ± 0.49^E^	1.48 ± 0.78^D^	2.12 ± 1.06^C^	3.48 ± 1.72^B^	4.07 ± 2.32^A^
MLE to menthol per session (mg)	3.01 ± 2.05^A^	3.01 ± 1.59^A^	2.89 ± 1.40^A^	2.39 ± 1.14^B^	2.04 ± 1.12^B^
MLE to aerosol per puff (mg)	4.98 ± 2.24^A^	5.13 ± 3.83^A^	4.99 ± 2.06^A^	4.65 ± 2.44^A^	4.49 ± 2.12^A^
MLE to nicotine per puff (mg)	0.03 ± 0.01^E^	0.05 ± 0.04^D^	0.08 ± 0.03^C^	0.14 ± 0.07^B^	0.18 ± 0.08^A^
MLE to menthol per puff (mg)	0.10 ± 0.05^A^	0.11 ± 0.08^A^	0.10 ± 0.04^A^	0.09 ± 0.05^A^	0.09 ± 0.04^A^

^1^Data are mean ± SD from 90 participants who used each product twice. Data were analysed by mixed-effects ANOVA, followed by Tukey’s post-hoc test. For a given parameter, values sharing the same alphabet letter were not significantly different (*p* > 0.05); those not sharing the same alphabet letter were significantly different (*p* < 0.05).

^2^Participants were given a maximum of 15-minutes in which to use the product.

When the participants used the products at home for 7 days, regular users of 47 mg/mL nicotine e-liquids puffed less frequently than the 18 and 34 mg/mL users (IPI, 143.6 vs. 74.2–84.5 s; intersession interval, 32.2 vs. 14.3–19.9 min), similar to the CLT; however, there were no significant differences in the average puff duration among the three nicotine concentrations (Table 4). Number of puffs taken (per day and per session) were lowest for the 47 mg/mL nicotine strength, although the difference was not significant. There were also no significant differences in the number of sessions per day or MLE to nicotine or aerosol per day (Table 4).

**Table 4:** HUT: comparison of puffing topography and MLE by nicotine concentration when using Crisp Mint e-liquid over 7 days^1^.

Parameter	18 mg/mL (*n* = 12)	34 mg/mL (*n* = 33)	47 mg/mL (*n* = 34)
Puffs per day	115.5 ± 83.2^A^	136.9 ± 124.9^A^	84.5 ± 55.7^A^
Puff duration (s)	1.66 ± 0.56^A^	1.58 ± 0.71^A^	1.67 ± 0.86^A^
Interpuff interval (s)	74.2 ± 92.9^B^	84.5 ± 57.3^B^	143.6 ± 94.0^A^
Sessions per day	7.1 ± 4.4^A^	8.6 ± 6.6^A^	5.7 ± 3.1^A^
Puffs per session	15.1 ± 4.9^A^	14.3 ± 3.5^A^	13.9 ± 4.0^A^
Intersession interval (minutes)	14.3 ± 8.7^B^	19.9 ± 12.4^B^	32.2 ± 25.5^A^
MLE ACM per puff (mg)	2.69 ± 1.22^A^	2.52 ± 1.54^A^	2.71 ± 1.87^A^
MLE nicotine per puff (mg)	0.04 ± 0.02^B^	0.07 ± 0.05^B^	0.11 ± 0.08^A^
MLE menthol per puff (mg)	0.06 ± 0.02^A^	0.05 ± 0.03^A^	0.05 ± 0.04^A^
MLE ACM per day (mg)	307.8 ± 237.4^A^	339.5 ± 361.1^A^	220.0 ± 201.0^A^
MLE nicotine per day (mg)	4.76 ± 3.65^A^	9.73 ± 10.55^A^	8.70 ± 8.19^A^
MLE menthol per day (mg)	6.61 ± 5.03^A^	6.92 ± 7.32^A^	4.40 ± 4.04^A^

^1^ Participants used only their usual nicotine concentration (18, 34 or 47 mg/mL) during home use testing. Only participants with valid data are included in the table. Data were analysed by one-way ANOVA, followed by Tukey’s post-hoc test. For a given parameter, values sharing the same alphabet letter were not significantly different (*p* > 0.05); values not sharing the same alphabet letter were significantly different (*p* < 0.05).

Overall, the trends observed in the CLT and HUT suggest that ENDS users partially compensate for higher nicotine and adapt their puffing behaviour, by changing either their puff duration or IPI (and hence number of puffs), in response to e-liquid nicotine strength to maintain a preferred level of nicotine exposure.

#### Effect of e-liquid flavour

We also determined whether a consumer’s puffing topography and estimated MLE change depending on the e-liquid flavour, by both CLT, where all participants (*n* = 90) used four different flavours at 18 mg/mL nicotine, and HUT, where these four products were used only by the regular users of 18 mg/mL nicotine products (*n* = 19).

When using the Golden Tobacco e-liquid during CLT, participants took fewer puffs of shorter duration and puffed less frequently as compared with the Crisp Mint, Peppermint Tobacco and Tropical Mango e-liquids (24.1 vs. 30.5–31.4 puffs per session, 2.13 vs. 2.27–2.59 s puff duration, and 46.6 vs. 33.6–35.3s IPI); they also took slightly larger puffs of longer duration when using the Tropical Mango e-liquid (55.2 vs. 46.2–49.5mL, 2.59 vs. 2.13–2.32s) (Table 5). Consequently, the MLE to aerosol mass and nicotine per session was lower for the Golden Tobacco e-liquid as compared with the other flavours (105.9 vs. 130.9–158.5 mg aerosol and 1.64 vs. 2.12–3.10 mg nicotine, per session). This might be attributable to the low Overall liking rating for this product, which was scored, on average, 2.0 on a scale of 1–5, where 1 was “dislike a lot” and 5 was “like a lot” (Supplementary Table S3). In comparison, the average Overall liking rating for the other flavours were 3.3, 3.5 and 3.7 for Peppermint Tobacco, Crisp Mint and Tropical Mango, respectively.

**Table 5:** CLT: comparison of puffing topography and MLE by flavour at 18 mg/mL nicotine^1^.

Parameter	Crisp Mint	Golden tobacco	Peppermint tobacco	Tropical mango
Puff number^2^	30.5 ± 18.0^A^	24.1 ± 17.0^B^	31.3 ± 17.3^A^	31.4 ± 16.1^A^
Puff volume (mL)	49.5 ± 19.9^B^	46.2 ± 21.7^B^	48.0 ± 19.4^B^	55.2 ± 23.8^A^
Puff duration (s)	2.27 ± 0.89^BC^	2.13 ± 0.90^C^	2.32 ± 0.91^B^	2.59 ± 1.12^A^
Interpuff interval (s)	35.3 ± 21.1^B^	46.6 ± 39.8^A^	33.6 ± 16.6^B^	33.9 ± 22.7^B^
Effort (cmWGs)	812 ± 602^B^	675 ± 607^C^	920 ± 624^A^	1002 ± 705^A^
MLE to aerosol per session (mg)	139.8 ± 70.6^B^	105.9 ± 78.3^C^	130.9 ± 70.9^B^	158.5 ± 98.1^A^
MLE to nicotine per session (mg)	2.12 ± 1.06^B^	1.64 ± 1.18^C^	3.10 ± 1.65^A^	2.40 ± 1.48^B^
MLE to menthol per session (mg)^3^	2.89 ± 1.40^A^	–	3.09 ± 1.61^A^	–
MLE to aerosol per puff (mg)	4.99 ± 2.06^A^	5.55 ± 5.22^A^	4.82 ± 3.26^A^	5.30 ± 2.15^A^
MLE to nicotine per puff (mg)	0.08 ± 0.03^B^	0.09 ± 0.08^B^	0.12 ± 0.08^A^	0.08 ± 0.03^B^
MLE to menthol per puff (mg)^d^	0.10 ± 0.04^A^	–	0.12 ± 0.08^A^	–

^1^Data are mean ± SD from 90 participants who used each product twice. Data were analysed by mixed-effects ANOVA, followed by Tukey’s post-hoc test. For a given parameter, values sharing the same alphabet letter were not significantly different (*p* > 0.05); those not sharing the same alphabet letter were significantly different (*p* < 0.05).

^2^Participants were given a maximum of 15-minutes in which to use the product.

^3^Only products containing menthol.

In the HUT, some differences in puffing topography parameters were noted, but there were no significant differences in MLE to aerosol mass or nicotine per day among the flavours, suggesting that flavour may have little effect on MLE per day when these products are used in a naturalistic environment (Table 6). It is worth noting that the overall sample size of 18 mg/mL users was relatively low (*n* = 19) for this HUT. In addition, valid data were collected for only 12 participants using Crisp Mint, 18 using Golden Tobacco, 11 using Peppermint Tobacco and 16 using Tropical Mango because some participants chose not to use certain flavoured pods.

**Table 6:** HUT: comparison of puffing topography and MLE by flavour at 18 mg/mL nicotine^1^.

Parameter	Crisp Mint (*n* = 12)	Golden tobacco (*n* = 18)	Peppermint tobacco (*n* = 11)	Tropical mango (*n* = 16)
Puffs per day	115.5 ± 83.2^A^	57.7 ± 44.3^B^	67.9 ± 66.0^AB^	74.2 ± 60.5^AB^
Puff duration (s)	1.66 ± 0.56^AB^	1.51 ± 0.74^B^	1.82 ± 0.76^AB^	1.95 ± 0.86^A^
Interpuff interval (s)	74.2 ± 92.9^A^	112.1 ± 67.4^A^	102.6 ± 156.0^A^	76.4 ± 60.4^A^
Sessions per day	7.1 ± 4.4^A^	4.4 ± 2.3^A^	5.1 ± 3.3^A^	5.2 ± 2.8^A^
Puffs per session	15.1 ± 4.9^A^	11.3 ± 4.8^AB^	10.4 ± 5.7^B^	12.0 ± 5.4^AB^
Intersession interval (minutes)	14.3 ± 8.7^AB^	21.5 ± 14.3^A^	13.8 ± 12.4^B^	14.7 ± 10.0^AB^
MLE ACM per puff (mg)	2.69 ± 1.22^AB^	2.38 ± 1.61^B^	3.03 ± 1.65^AB^	3.34 ± 1.86^A^
MLE nicotine per puff (mg)	0.04 ± 0.02^B^	0.04 ± 0.02^B^	0.07 ± 0.04^A^	0.05 ± 0.03^B^
MLE menthol per puff (mg)^2^	0.06 ± 0.02^A^	–	0.07 ± 0.04^A^	–
MLE ACM per day (mg)	307.8 ± 237.4^A^	167.1 ± 201.4^A^	261.6 ± 328.6^A^	282.0 ± 325.7^A^
MLE nicotine per day (mg)	4.76 ± 3.65^A^	2.62 ± 3.09^A^	6.24 ± 7.78^A^	4.30 ± 4.94^A^
MLE menthol per day (mg)	6.61 ± 5.03^A^	–	6.25 ± 7.74^A^	–

^1^Used among participants who were regular users of 18 mg/mL nicotine e-liquid. Only those with valid data are included in the table. Data were analysed by mixed-effects ANOVA, followed by Tukey’s post-hoc test. For a given parameter, values sharing the same alphabet letter were not significantly different (*p* > 0.05); those not sharing the same alphabet letter were significantly different (*p* < 0.05).

^2^Only products containing menthol.

#### Comparison with a tobacco cigarette

Lastly, we compared vapers’ MLE to nicotine when using the 47 mg/mL ENDS versus smokers’ MLE when smoking a 15-mg ISO tar cigarette. The MLE to nicotine per puff for the 47 mg/mL ENDS was significantly lower than that from the cigarette (0.18 vs. 0.27 mg per puff; t test, *p* < 0.0001). During the 15-minutes session, however, the ENDS users took significantly more puffs than the smokers (24.9 vs. 16.9 puffs; *p* < 0.0001); thus, the total MLE to nicotine for the puffing session was similar between the two products (4.07 mg per 15-minutes vaping session vs. 4.52 mg per cigarette; *p* = 0.3307).

### Self-reported average daily consumption

During the HUT, participants self-reported their consumption of e-liquid cartridges via consumption diaries that were completed daily (Table 7). ADC ranged from 0.55 (Peppermint Tobacco) to 0.75 (Crisp Mint) cartridge per day. There were no significant differences observed in the ADC among the products.

**Table 7:** HUT: Self-reported ADC of the study products over 7-days home use^1^.

Product	No. of users	ADC (cartridges per day)
18 mg/mL nicotine users		
Crisp Mint	15	0.75 ± 0.49^A^
Golden tobacco	16	0.59 ± 0.99^A^
Peppermint tobacco	12	0.55 ± 0.38^A^
Tropical mango	15	0.58 ± 0.33^A^
34 mg/mL nicotine users		
Crisp Mint	31	0.74 ± 0.61^A^
47 mg/mL users		
Crisp Mint 47 mg/mL	32	0.58 ± 0.36^A^

^1^Data were self-reported via a consumption diary completed daily. Data were analysed by mixed-effects ANOVA, followed by Tukey’s post-hoc test. For a given parameter, values sharing the same alphabet letter were not significantly different (*p* > 0.05); those not sharing the same alphabet letter were significantly different (*p* < 0.05).

## Discussion

The present study has evaluated whether puffing topography and MLE to nicotine and aerosol alters with changes in e-liquid nicotine concentration and flavour type. In particular, we hypothesized that users would demonstrate compensatory puffing such that daily exposure to nicotine would remain the same regardless of the nicotine concentration of the e-liquid, as established in cigarette smoking^43^ and flavours would have little effect if any.

In the CLT, where puffing topography was measured during a fixed period of time (15-minutes), puff number and duration generally decreased, while IPI increased as the nicotine concentration increased from 6 to 47 mg/mL, suggesting that participants altered their puffing behaviour in an attempt to compensate for the higher concentration of nicotine. Consistent with partial compensation, MLE to nicotine increased with nicotine concentration, while MLE to aerosol and menthol decreased in keeping with the reduced frequency of puffing. Notably, in the HUT, users of the highest nicotine concentration e-liquid (47 mg/mL) showed no significant differences in puff duration or number of puffs taken as compared with the users of 18 and 34 mg/mL nicotine; however, they puffed less frequently to achieve the same MLE per day to nicotine and aerosol, in support of our hypothesis.

To investigate whether attempts at compensatory puffing differed according to participants’ usual nicotine concentration, we also stratified the CLT data by regular-use nicotine group (18, 34, and 47 mg/mL), as presented in Supplementary Table S2. Across all groups, puff number, puff duration, and MLE to aerosol per session generally decreased with increasing study product nicotine concentration, while MLE to nicotine (per session and per puff) increased. Although absolute values differed modestly among groups, the direction and magnitude of compensatory behaviour were broadly similar. These findings suggest that partial compensatory puffing is a general behavioural response rather than one specific to any particular habitual nicotine-use level. Since the study was not powered for between-group comparisons, numerical differences among regular-use nicotine groups should not be interpreted as meaningful.

The influence of nicotine concentration on ENDS puffing behaviour observed in the present study is broadly consistent with other published literature demonstrating partial compensatory use when nicotine concentration is reduced. An early topography study (*n* = 32 participants) similarly noted that a low nicotine (3 mg/mL) e-liquid resulted in longer puffs at increased frequency, higher e-liquid consumption and lower nicotine consumption, as compared with higher nicotine (8 mg/mL), suggesting that participants did not fully compensate in response to nicotine concentration^37^. A controlled 10-puff study of 33 experienced e-cigarette users and 31 smokers showed a moderate increase in puff duration for low nicotine e-liquid (zero nicotine and 8 mg/mL) compared with higher nicotine e-liquid (18 and 36 mg/mL), while also showing a moderate increase puff duration for 18 mg/mL compared to 36 mg/mL among smokers, but not for experienced e-cigarette users^42^. Similarly, a small clinical study of 11 participants using 6 and 24 mg/mL nicotine e-liquids reported that liquid consumption and puff number were higher, and puff duration longer, in the low nicotine strength condition, with higher plasma nicotine levels in the high nicotine strength condition^41^. A follow-on study with 20 participants also found longer puff duration, increased puff number, shorter IPI and greater e-liquid consumption when using 6 mg/mL nicotine compared with 18 mg/mL nicotine^40^. In contrast, a more recent study of 61 participants using a connected device in a naturalistic environment reported that higher nicotine concentrations led to comparable puff durations, as well as significantly higher nicotine consumption, compared to low nicotine concentrations^31^; in this study participants self-selected the device power level (7–13 W) and nicotine concentration (0–36 mg/mL). As noted by the author, this contrast in findings compared to other research^37,40–42^ may be due to complex interactions between device power and nicotine concentration, where some users may prefer to use certain power settings for a certain range of nicotine concentrations, such as high power with low nicotine or vice versa, or high power with high nicotine and thus device power may have confounded any effect of nicotine concentration on their puffing behaviour^31^.

Collectively, these studies show that ENDS users engage in compensatory behaviour when using lower nicotine concentrations in order to achieve a preferred level of nicotine intake, typically through longer or more frequent puffs, and this leads to higher consumption of e-liquid. This may in turn lead to higher consumption of aerosol constituents, other than nicotine which as discussed has been shown to be higher in high nicotine e-liquids compared to low nicotine e-liquids. Our findings extend this pattern to a larger sample and a wider range of five nicotine concentrations (6–47 mg/mL), in both controlled (CLT) and real-world (HUT) settings.

In terms of flavour, in the CLT, participants took significantly fewer puffs (24 vs. 30–31) of shorter duration (2.1 vs. 2.3–2.6) and puffed less frequently (IPI, 47 vs. 34–35 s) when using the Golden Tobacco e-liquid as compared with the other flavours. The reduced puff durations and frequency corresponded to lower MLE to nicotine and aerosol for the Golden Tobacco flavour, consistent with the substantially lower rating for Overall liking (average score 2.0) compared to the other flavours (3.3–3.7).

Other studies have also noted lower puffing parameters for tobacco-flavoured e-liquids compared to mint or fruit flavours. For example, mint/menthol flavours have been associated with higher puffing intensity, higher plasma nicotine and greater satisfaction than tobacco flavour^47^; and strawberry flavour produced longer puff durations and greater e-liquid consumption (3.2 s; 316 mg) than tobacco flavour (2.8 s; 242 mg), consistent with higher preference for the strawberry flavour, with even more intensive puffing observed on participants’ preferred usual brand e-liquid flavour^38^. Similarly, in a topography and nicotine pharmacokinetics (PK) study of five flavours, the shortest puff durations (2.8 s) and lowest satisfaction were observed for tobacco, and the longest puff durations (3.3 s) and highest satisfaction for menthol^39^. In contrast a 2-week naturalistic study, where participants used a tobacco flavour and either a berry or menthol flavour for one week each at their preferred nicotine level (6, 12 or 18 mg/mL), reported that flavour affected puff flow rate and puff volume, but not puff duration, puff interval or consumption; the direction of the flavour effect varied across individuals, with no single flavour showing a consistent pattern^32^. In contrast, one study found no difference in puffing topography or plasma nicotine when comparing a tobacco flavour with participants’ preferred flavour, despite higher liking for the preferred flavour^48^. Although these studies, and our own results, demonstrate that flavour can influence puffing behaviour and aerosol intake, this does not necessarily translate into meaningful differences in systemic nicotine exposure in controlled PK studies. For example, a study of four Vuse Solo flavours^49^ and a study of four Vuse Alto flavours^50^ observed similar nicotine PK profiles across flavour variants, which included tobacco, mint and berry flavours. Likewise, Voos et al., reported that flavour-related differences in puff duration did not correspond to differences in plasma nicotine, which was highest for cherry flavour and likely influenced by other factors such as e-liquid pH, as suggested in other research^38,51^. It should be noted that PK studies typically involve 12-hour nicotine abstinence prior to product use, and a single short (~ 10-minute) use period^49,50^, conditions that may limit the comparison of PK studies to puffing topography studies.

Interestingly, in the present study’s HUT of e-liquid flavours, although not statistically significant, the relatively higher number of puffs taken and higher MLE to aerosol per day when using the Crisp Mint e-liquid compared with using the other flavours (115.5 vs. 57.7–74.2 puffs per day and 307.8 vs. 167.1–282.0 mg aerosol per day) may be attributable to the accessibility of this flavour. In the New Zealand market, only tobacco and mint flavoured e-liquids can be sold in convenience stores, while fruit flavoured e-liquids can be purchased only from specialist vape shops^52^. Collectively, the data from the flavour comparison indicate that flavour likeability is important in the use of ENDS, and may have a role to play in encouraging smokers to switch from tobacco cigarettes to these products.

To be successful in a THR approach, ENDS must be able to deliver nicotine efficiently as well as meeting the sensory needs of consumers. In the CLT comparison of puffing topography and MLE between smokers and ENDS users (47 mg/mL nicotine), the two groups of participants attained a similar MLE to nicotine, indicating that nicotine provision from ENDS is likely to be adequate for smokers. Smokers switching to ENDS will favour products with a nicotine concentration that provides effects similar to those they derive from smoking; in this respect, the availability of e-liquids with different nicotine concentrations will be an important component of a THR approach. However, while pharmacological effects of nicotine are important, other factors including device draw resistance, sensory effects and conditioned stimuli may also contribute to smokers’ initial switch and continued use of ENDS.

The present study has some strengths. To our knowledge, it is one of the largest puffing topography studies involving 90 regular ENDS consumers using all eight e-liquids in the CLT. In addition, measurements were taken both under fixed conditions at a central location and during home use in a real-world situation. Lastly, a range of nicotine concentrations was compared (*n* = 5), whereas previous studies have typically compared only “high” and “low” concentrations.

The study also has some limitations. The CLT provided a snapshot of use behaviour over 15-minutes among participants using the ENDS through an unfamiliar puffing instrument and the data may not reflect real-life puffing behaviour; however, our findings were consistent with real-world data obtained by the HUT. In addition, in the comparison of flavoured e-liquids at home, the sample size was limited to only those participants who normally used a nicotine strength of 18 mg/mL.

In summary, both level of nicotine and flavour likeability play an important role in consumers’ puffing behaviour and MLE to nicotine and aerosol; therefore, it is important to be able to offer smokers a variety of nicotine concentrations and flavours to increase the likelihood that ENDS can provide a satisfactory alternative to continued smoking for smokers who would not otherwise quit. Among our cohort, daily exposure to nicotine remained the same regardless of the nicotine concentration of the e-liquid, because users altered their puffing behaviour to achieve a preferred level of nicotine. It is also clear that the higher the level of nicotine in the e-liquid, the lower a consumer’s MLE to aerosol and thus to other aerosol constituents such as propylene glycol, vegetable glycerin and flavours. Further work to understand how the combination of e-liquid and device parameters affect the amount and content of aerosol generated is needed to fully elucidate the role of varying nicotine concentrations.

## Supplementary Information

Below is the link to the electronic supplementary material.

Supplementary Material 1

Supplementary Material 2

## Data Availability

The datasets used and/or analysed during the current study are available from the corresponding author on reasonable request.
