# Supplementary material for: Influence of nicotine concentration and flavours on mouth level exposure and puffing topography among regular e-cigarette consumers in New Zealand
Source: Sci Rep. 2026 May 30;16:24843. doi: 10.1038/s41598-026-53889-3 (PMC13457872; doi:10.1038/s41598-026-53889-3)
Supplement: Supplementary file 1 [file 41598_2026_53889_MOESM1_ESM.docx]

**Supplementary Information**

**Supplementary Figure S1.** Calibration of the PUB device. Top, fitted line plot of mean puff duration measured by PUB device vs collected aerosol mass (ACM). Bottom, scatter plot of estimated ACM per puff vs actual ACM per puff.

**Supplementary Table S1.** Sensory perception responses by nicotine level for Crisp Mint e-liquid ^1^.

| **Sensory attribute** | **6 mg/mL (n=83)** | **12 mg/mL (n=68)** | **18 mg/mL (n=72)** | **34 mg/mL (n=75)** | **47 mg/mL (n=84)** |
| --- | --- | --- | --- | --- | --- |
| Overall liking | 3.5 ± 0.9 ^A^ | 3.4 ± 1.0 ^A^ | 3.5 ± 0.9 ^A^ | 3.3 ± 0.9 ^A^ | 3.4 ± 1.0 ^A^ |
| Immediate aerosol delivery | 3.3 ± 1.0 ^A^ | 3.4 ± 0.8 ^A^ | 3.3 ± 0.8 ^A^ | 3.4 ± 0.7 ^A^ | 3.4 ± 0.7 ^A^ |
| Mouth filling | 2.9 ± 0.9 ^B^ | 3.2 ± 08 ^AB^ | 3.1 ± 0.8 ^AB^ | 3.1 ± 0.6 ^AB^ | 3.2 ± 0.8 ^A^ |
| Taste amount | 3.2 ± 1.1 ^A^ | 3.3 ± 0.9 ^A^ | 3.4 ± 0.9 ^A^ | 3.5 ± 0.8 ^A^ | 3.4 ± 0.8 ^A^ |
| Draw effort | 3.1 ± 1.1 ^A^ | 3.1 ± 0.9 ^A^ | 3.0 ± 0.8 ^A^ | 3.1 ± 0.7 ^A^ | 3.0 ± 0.8 ^A^ |
| Intensity | 2.3 ± 1.0 ^C^ | 3.0 ± 1.0 ^B^ | 2.9 ± 0.9 ^B^ | 3.2 ± 0.8 ^B^ | 3.6 ± 0.8 ^A^ |
| Irritation | 1.8 ± 1.0 ^C^ | 2.5 ± 1.0 ^B^ | 2.5 ± 1.0 ^B^ | 2.8 ± 1.0 ^AB^ | 3.0 ± 1.1 ^A^ |
| Taste liking | 3.3 ± 1.1 ^A^ | 3.2 ± 1.1 ^A^ | 3.4 ± 1.0 ^A^ | 3.2 ± 1.1 ^A^ | 3.3 ± 1.1 ^A^ |
| ^1^ Participants were asked to rate sensory attributes on a scale of 1 – 5, where 1 was ‘Low’ and 5 was ‘High’ (for Overall liking and Taste liking only: 1 was ‘Dislike a lot’ and 5 was ‘Like a lot’). Data were analysed by mixed-effects ANOVA, followed by Tukey’s post hoc-test. For a given attribute, values sharing the same alphabet letter were not significantly different (p>0.05); those not sharing the same alphabet letter were significantly different (p<0.05). | | | | | |

**Supplementary Table S2.** Puffing topography and MLE by nicotine concentration, per regular use nicotine concentration ^1^.

| **Topography & MLE attribute** | **Nicotine User Group** | **Study Product Nicotine Concentration** | | | | |
| --- | --- | --- | --- | --- | --- | --- |
|  |  | **6 mg/mL** | **12 mg/mL** | **18 mg/mL** | **34 mg/mL** | **47 mg/mL** |
| Puff Count | 18 mg/mL users | 27.6 ± 11.0 | 32.8 ± 15.0 | 27.2 ± 13.6 | 23.3 ± 10.5 | 21.2 ± 10.5 |
|  | 34 mg/mL users | 31.8 ± 14.0 | 32.4 ± 13.0 | 31.0 ± 17.3 | 29.8 ± 18.8 | 25.3 ± 13.7 |
|  | 47 mg/mL users | 30.3 ± 18.3 | 31.6 ± 17.7 | 31.8 ± 20.6 | 30.1 ± 16.4 | 26.4 ± 14.3 |
| Puff Volume (mL) | 18 mg/mL users | 46.9 ± 18.5 | 43.7 ± 17.9 | 42.7 ± 17.1 | 44.0 ± 15.7 | 38.2 ± 13.6 |
|  | 34 mg/mL users | 55.0 ± 25.5 | 54.4 ± 21.9 | 51.5 ± 22.2 | 53.2 ± 22.3 | 50.0 ± 22.9 |
|  | 47 mg/mL users | 52.7 ± 20.5 | 48.7 ± 18.1 | 51.2 ± 18.8 | 50.4 ± 18.1 | 45.2 ± 17.8 |
| Puff Duration (s) | 18 mg/mL users | 2.58 ± 0.95 | 2.32 ± 1.03 | 2.10 ± 0.84 | 2.10 ± 0.74 | 2.02 ± 0.71 |
|  | 34 mg/mL users | 2.57 ± 0.86 | 2.34 ± 0.65 | 2.27 ± 0.75 | 2.19 ± 0.60 | 2.10 ± 0.65 |
|  | 47 mg/mL users | 2.59 ± 1.00 | 2.35 ± 0.97 | 2.35 ± 1.04 | 2.21 ± 0.95 | 2.14 ± 0.93 |
| Interpuff Interval (s) | 18 mg/mL users | 36.6 ± 16.2 | 30.8 ± 17.0 | 40.7 ± 19.7 | 43.6 ± 21.5 | 47.2 ± 17.7 |
|  | 34 mg/mL users | 31.1 ± 14.0 | 30.6 ± 15.0 | 32.6 ± 15.3 | 35.3 ± 15.8 | 38.5 ± 16.1 |
|  | 47 mg/mL users | 37.9 ± 27.5 | 43.9 ± 54.8 | 35.1 ± 25.8 | 39.1 ± 28.2 | 46.0 ± 35.8 |
| Effort (cmWGs) | 18 mg/mL users | 791 ± 534 | 728 ± 457 | 582 ± 404 | 496 ± 206 | 424 ± 347 |
|  | 34 mg/mL users | 994 ± 518 | 942 ± 416 | 851 ± 496 | 833 ± 517 | 653 ± 317 |
|  | 47 mg/mL users | 998 ± 895 | 874 ± 660 | 894 ± 743 | 773 ± 474 | 675 ± 532 |
| MLE to aerosol per session (mg) | 18 mg/mL users | 128.0 ± 78.9 | 152.7 ± 102.3 | 118.7 ± 59.4 | 94.3 ± 49.7 | 91.5 ± 58.6 |
|  | 34 mg/mL users | 160.1 ± 117.7 | 144.9 ± 65.0 | 142.7 ± 70.9 | 125.1 ± 51.0 | 110.1 ± 67.6 |
|  | 47 mg/mL users | 147.1 ± 95.6 | 146.3 ± 82.6 | 147.9 ± 75.1 | 124.4 ± 62.9 | 98.6 ± 40.9 |
| MLE to nicotine per session (mg) | 18 mg/mL users | 0.65 ± 0.38 | 1.53 ± 1.00 | 1.81 ± 0.89 | 2.76 ± 1.51 | 3.66 ± 2.43 |
|  | 34 mg/mL users | 0.80 ± 0.56 | 1.46 ± 0.63 | 2.17 ± 1.06 | 3.69 ± 1.54 | 4.43 ± 2.80 |
|  | 47 mg/mL users | 0.74 ± 0.46 | 1.47 ± 0.81 | 2.24 ± 1.12 | 3.67 ± 1.90 | 3.95 ± 1.70 |
| MLE to menthol per session (mg) | 18 mg/mL users | 2.60 ± 1.60 | 3.12 ± 2.03 | 2.47 ± 1.18 | 1.91 ± 1.00 | 1.84 ± 1.18 |
|  | 34 mg/mL users | 3.25 ± 2.38 | 2.96 ± 1.29 | 2.95 ± 1.41 | 2.53 ± 1.02 | 2.21 ± 1.36 |
|  | 47 mg/mL users | 2.99 ± 1.94 | 2.99 ± 1.64 | 3.05 ± 1.49 | 2.51 ± 1.26 | 1.98 ± 0.82 |
| MLE to aerosol per puff (mg) | 18 mg/mL users | 4.57 ± 2.07 | 4.72 ± 2.38 | 4.59 ± 1.91 | 4.32 ± 2.47 | 4.73 ± 2.75 |
|  | 34 mg/mL users | 5.10 ± 2.54 | 4.77 ± 1.56 | 4.98 ± 1.89 | 5.07 ± 2.92 | 4.52 ± 1.75 |
|  | 47 mg/mL users | 5.08 ± 2.07 | 5.67 ± 5.53 | 5.20 ± 2.29 | 4.44 ± 1.88 | 4.33 ± 2.11 |
| MLE to nicotine per puff (mg) | 18 mg/mL users | 0.02 ± 0.01 | 0.05 ± 0.02 | 0.07 ± 0.03 | 0.13 ± 0.07 | 0.19 ± 0.11 |
|  | 34 mg/mL users | 0.03 ± 0.01 | 0.05 ± 0.02 | 0.08 ± 0.03 | 0.15 ± 0.09 | 0.18 ± 0.07 |
|  | 47 mg/mL users | 0.03 ± 0.01 | 0.06 ± 0.06 | 0.08 ± 0.03 | 0.13 ± 0.06 | 0.17 ± 0.08 |
| MLE to menthol per puff (mg) | 18 mg/mL users | 0.09 ± 0.04 | 0.10 ± 0.05 | 0.10 ± 0.04 | 0.09 ± 0.05 | 0.09 ± 0.06 |
|  | 34 mg/mL users | 0.10 ± 0.05 | 0.10 ± 0.03 | 0.10 ± 0.04 | 0.10 ± 0.06 | 0.09 ± 0.04 |
|  | 47 mg/mL users | 0.10 ± 0.04 | 0.12 ± 0.11 | 0.11 ± 0.05 | 0.09 ± 0.04 | 0.09 ± 0.04 |
| ^1^ Data are mean ± SD of n=19 for 18 mg/mL users, n=34 for 34 mg/mL users and n=37 for 47 mg/mL users. Results are descriptive only.  ^2^ Participants were given a maximum of 15-minutes in which to use the product. | | | | | | |

**Supplementary Table S3.** Sensory perception responses by flavour at a nicotine concentration of 18 mg/mL ^1^.

| **Attribute** | **Crisp Mint**  **(n=72)** | **Golden Tobacco (n=85)** | **Peppermint Tobacco (n=81)** | **Tropical Mango (n=86)** |
| --- | --- | --- | --- | --- |
| Overall liking | 3.5 ± 0.9 ^A^ | 2.0 ± 1.0 ^B^ | 3.3 ± 1.0 ^A^ | 3.7 ± 1.1 ^A^ |
| Immediate aerosol delivery | 3.3 ± 0.8 ^A^ | 3.0 ± 0.9 ^B^ | 3.3 ± 0.8 ^A^ | 3.2 ± 1.0 ^AB^ |
| Mouth filling | 3.1 ± 0.8 ^A^ | 2.8 ± 0.8 ^B^ | 3.2 ± 0.6 ^A^ | 2.9 ± 0.9 ^AB^ |
| Taste amount | 3.4 ± 0.9 ^A^ | 3.3 ± 1.1 ^A^ | 3.4 ± 0.7 ^A^ | 3.4 ± 1.0 ^A^ |
| Draw effort | 3.0 ± 0.8 ^A^ | 3.0 ± 1.0 ^A^ | 3.0 ± 0.9 ^A^ | 3.1 ± 1.0 ^A^ |
| Intensity | 2.9 ± 0.9 ^A^ | 2.9 ± 1.1 ^A^ | 3.0 ± 0.8 ^A^ | 2.6 ± 1.0 ^B^ |
| Irritation | 2.5 ± 1.0 ^AB^ | 2.8 ± 1.2 ^A^ | 2.6 ± 0.9 ^AB^ | 2.4 ± 1.1 ^B^ |
| Taste liking | 3.4 ± 1.0 ^B^ | 1.8 ± 1.0 ^C^ | 3.2 ± 1.0 ^B^ | 3.8 ± 1.1 ^A^ |
| ^1^ Participants were asked to rate sensory attributes on a scale of 1 – 5, where 1 was ‘Low’ and 5 was ‘High’ (for Overall liking and Taste liking only: 1 was ‘Dislike a lot’ and 5 was ‘Like a lot’). Data were analysed by mixed-effects ANOVA, followed by Tukey’s post-hoc test. For a given attribute, values sharing the same alphabet letter were not significantly different (p>0.05); those not sharing the same alphabet letter were significantly different (p<0.05). | | | | |
